# Therapeutic potential of human mesenchymal stromal cell-derived mitochondria in a rat model of surgical digestive fistula

**DOI:** 10.1038/s41598-025-13887-3

**Published:** 2025-08-09

**Authors:** Antoine Mariani, Augustin Guichard, Anna C. Sebbagh, André Cronemberger Andrade, Zahra Al Amir Dache, Christopher Ribes, Dmitry Ayollo, Mehdi Karoui, Gregory Lavieu, Florence Gazeau, Amanda K. A. Silva, Gabriel Rahmi, Sabah Mozafari

**Affiliations:** 1 Assistance Publique-Hôpitaux de Paris, Service de Chirurgie Digestive et Oncologique, Hôpital Européen Georges Pompidou, Paris, 75015 France; 2 Université Paris Cité, CNRS UMR8175, INSERM U1334, Laboratory NABI (Nanomédecine, Biologie Extracellulaire, Intégratome et Innovations en santé), Paris, 75006 France; 3 Assistance Publique-Hôpitaux de Paris, Service d’hépato-gastro-entérologie et endoscopies digestives, Hôpital Européen Georges Pompidou, Paris, 75015 France; 4 Department of Clinical Neurosciences and National Institute for Health Research (NIHR) Biomedical Research Centre, University of Cambridge, Cambridge, CB2 0AH UK

**Keywords:** Mitochondria transplantation, Post-surgical fistula, Human mesenchymal stromal cells (hMSCs), Biotherapy, Wound healing, regenerative medicine, Stem cells, Gastroenterology

## Abstract

Mitochondria are central to cellular energy metabolism and play a critical role in tissue regeneration. Mitochondrial dysfunction contributes to a range of degenerative conditions and impaired wound healing, driving increasing interest in mitochondrial transplantation as a novel therapeutic strategy. Gastrointestinal wound healing is particularly susceptible to failure, with complications such as post-surgical fistula formation commonly occurring after procedures like sleeve gastrectomy. Mitochondria derived from human mesenchymal stromal/stem cells (hMSCs) have shown promise in restoring tissue bioenergetics and promoting repair across various disease models. In this study, we evaluated the therapeutic potential of hMSC-derived mitochondria as a nano-biotherapy for gastrointestinal wound healing using a rat model of post-operative fistula. Structurally intact mitochondria were isolated from hMSCs and either applied to human colonic epithelial cells (HCEC-1CT) in vitro or transplanted locally into fistula-bearing rats. Mitochondrial treatment led to a dose-dependent increase in cellular metabolic activity, intracellular ATP levels, and mitochondrial uptake by recipient cells. In vivo, mitochondrial transplantation significantly accelerated fistula closure and tissue regeneration compared to controls. These findings underscore the translational promise of mitochondria-based, cell-free therapies and lay the groundwork for future regenerative strategies targeting gastrointestinal wound repair.

**Supplementary Information:**

The online version contains supplementary material available at 10.1038/s41598-025-13887-3.

## Introduction

Effective tissue regeneration is an energy-intensive process critically dependent on mitochondrial function^1,2^. As the primary source of cellular ATP and key regulators of metabolic signaling, mitochondria are central to wound healing, especially in high-turnover tissues like the gastrointestinal (GI) tract^3–6^. Impaired mitochondrial function has been linked to defective epithelial regeneration and chronic wound formation in the intestine^6–9^. Consequently, enhancing mitochondrial function in damaged tissues has emerged as a promising target for regenerative therapies.

Gostoperative digestive fistulas, particularly following sleeve gastrectomy, remain a serious clinical complication^10^ with incidences ranging from 1 to 5%^11^. These abnormal connections between GI organs or the skin can lead to severe infections, prolonged hospitalization, and even death (a mortality rate of 10% − 30%)^12^. Despite advancements in surgical techniques and supportive care, fistula management continues to pose significant therapeutic challenges. A recent study on 23 patients with postoperative enterocutaneous fistulas showed that while 70% of fistulas closed with a combination of sever conservative and surgical management, spontaneous closure occurred in only 17% of cases^10^. Current treatment strategies—including drainage, nutritional support, and endoscopic or surgical repair—often fail to fully restore tissue integrity^13–18^. Noori (2021) reported a 63% surgical success rate for proximal, high-output, or complex fistulas, with a mortality rate of 21.7% primarily due to sepsis, severe malnutrition, and organ failure^10^, underscoring the need for novel regenerative strategies to enhance healing and reduce complications. Different biotherapeutic strategies have suggested a promising avenue for tissue repair in this context recently. These include the use of mesenchymal stromal/stem cells (MSCs)^16,19,20^ or their secreted cell-free products such as extracellular vesicles (EVs)^21–23^ for intestinal fistula treatments. The regenerative and immunomodulatory potential of MSCs-based regenerative therapeutics has been increasingly attributed to the presence or transfer of metabolically active products, enzymes, or entire mitochondria^24–36^. Interestingly, analysis of MSCs-EVs revealed enrichment in proteins related to energy production and the presence of mitochondria^31,37^. Building on this concept, growing attention has turned to cell-free nano-biotherapies—such as MSC-derived mitochondria or EV-enriched mitochondria^38,39^, offering therapeutic benefits of cell therapies with lower risks of tumorigenesis, immune rejection, and vascular occlusion associated. Moreover, intercellular mitochondrial transfer has been proposed as a physiological mechanism for tissue revitalization, occurring via tunneling nanotubes, intercellular dendrites, vesicular cargo, or direct extrusion from donor cells followed by internalization by recipient cells^40,41^. Inspired by this natural process, direct mitochondrial transplantation has recently emerged as a novel therapeutic approach^42–44^. Notably, the clinical translation of mitochondrial transplantation is already underway. In a pilot clinical study involving pediatric patients with severe cardiogenic shock following ischemia–reperfusion injury, autologous mitochondrial transplantation was associated with successful separation from extracorporeal membrane oxygenation (ECMO) and improved ventricular function^45^. Several ongoing clinical trials using MSCs (as mitochondria cell source) and emerging biotech companies worldwide highlight the growing momentum and clinical potential of mitochondria-based therapeutics. While MSC-based mitochondrial transplantation has shown promise in various preclinical conditions—including the heart^46^, brain^47^, spinal cord^48^, lung^31^ and kidney^49^— its beneficial application in gastrointestinal wound healing remains largely unexplored. This represents a critical knowledge gap, particularly given the high energy demands of epithelial regeneration, the contribution of mitochondrial dysfunction to chronic digestive wound healing, and the significant clinical burden of post-surgical digestive fistulas. Here, we hypothesized that administering freshly isolated mitochondria could enhance the bioenergetic capacity of distressed intestinal cells in vitro and promote fistula closure by supporting tissue regeneration in vivo. To test this, we assessed the therapeutic efficacy of mitochondria derived from human adipose tissue MSCs using both in vitro models of metabolically stressed colonic cells and an in vivo rat model of post-operative digestive fistula.

## Results

### Viable and structurally intact mitochondria are isolated from hMSCs

**Fig. 1:**
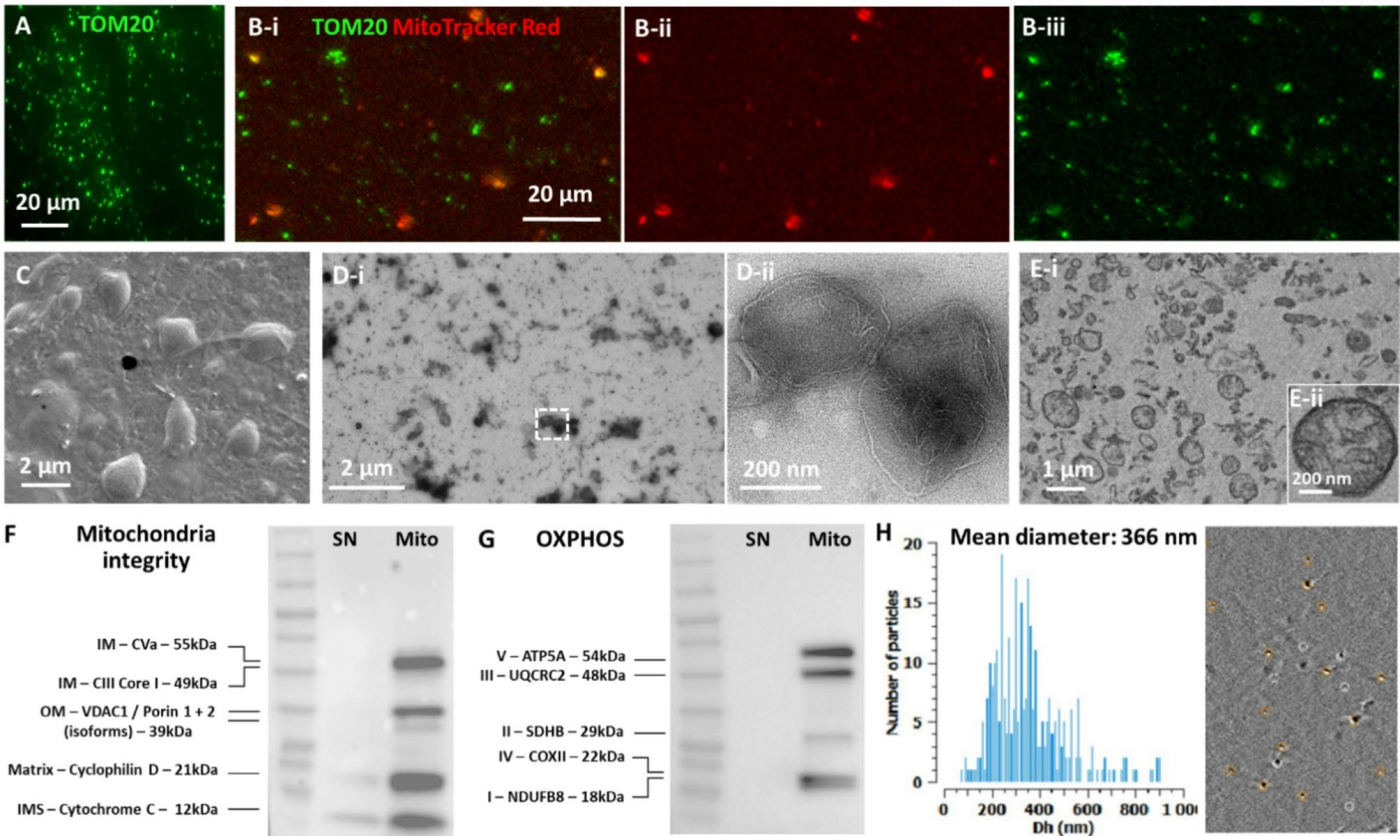
Characterization of human MSCs-derived mitochondria. (**A**) TOM20 (green) staining and (**Bi**–**Biii**) MitoTracker red/TOM20 colabling of freshly isolated mitochondria indicating isolation of numerous viable mitochondria from hMSCs. (**C**, **D**) Scanned electron microscopy (SEM) and transmission electron microscopy using negative staining (**Di**, **Dii**) as well as cross-sectioned methods (**Ei**, **Eii**) depicting structurally intact mitochondria of various diameter ranging from 100–1200 nm. (**F**, **G**) Quality control analysis of isolated mitochondria by Western blotting of mitochondria membrane integrity markers and human OXPHOS complexes (**GI**–**GV**) showing co-expression of mitochondrial structural and functional proteins in mitochondria preparations (Mito) compared to their corresponding supernatant (SN) loaded with the same amount of protein (Uncropped western blots can be found in the Supplementary Fig. 1). (**H**) ILM of mitochondria samples using Videodrop particle counter showing the size distribution graph with a mean size of 366 nm and a representative interferometry image (depicting the particles in white circles appeared for a moment or the tracked particles shown in circles with orange color). *IM* inner membrane, *OM* outer membrane, *IMS* intermembrane space.

The mitochondria from hMSCs were fully characterized through various assays. Immunofluorescence staining revealed robust expression of TOM20, a mitochondrial outer membrane marker (Fig. 1A). Further assessments through co-labeling with MitoTracker Red as well as TOM20 (Fig. 1Bi-iii) demonstrated the presence of viable mitochondria. Scanning electron microscopy (SEM) imaging showed multiple rounds to oval-shaped structures ranging from 0.2 to 2 μm in size with intact outer surfaces (Fig. 1C), corroborated by TEM analyses of negative staining or cross-sectioned methods depicting the presence of multiple mitochondria with inner membranes and a diverse range of diameters spanning from 0.1 to 1.2 μm (Fig. 1Di-Dii and Ei-Eii).

Quality control assessment of the isolated mitochondria was conducted through a series of Western blot analyses (Fig. 1F-G). For each preparation, the corresponding supernatant (SN) was used as a control to facilitate sample characterization. An equal amount of 20 µg protein was loaded for both the mitochondria and SN samples (Fig. 1F-G). The co-presence of five key mitochondrial membrane integrity markers (Fig. 1F), as well as five markers of the human oxidative phosphorylation (OXPHOS) complexes I-V in mitochondria preparations (a total of 10 markers), was confirmed by Western blot analysis for each independent mitochondria isolation experiment (Fig. 1G).

Mitochondrial size distribution analysis was performed using interferometric light microscopy (ILM) by videodrop particle counter. The mean size of isolated mitochondria was determined to be 366 nm, consistent with previous literature and TEM analysis (Fig. 1H).

### hMSC mitochondria promote the metabolic activity of starved colon cells in vitro

**Fig. 2:**
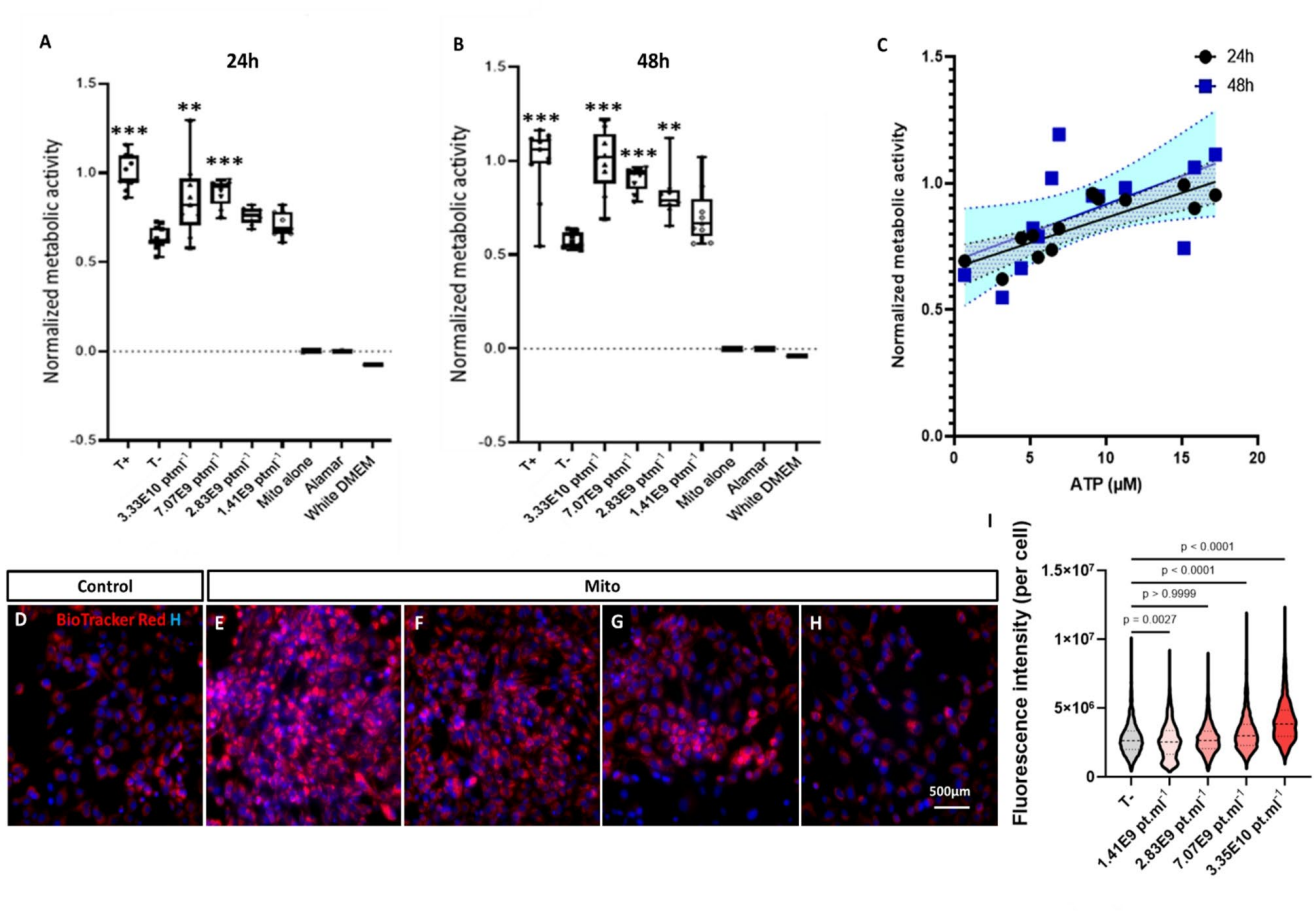
Metabolic activity of HCEC-1CT colonic cells following in vitro mitochondria transplantation. (**A**, **B**) Alamar results of the metabolic activity of cells exposed to fresh mitochondria at different doses compare to negative control (non-starved cells treated with RB) at 24 h (**A**) and 48 h (**B**) normalized to positive control (*n* = 13 wells per condition, 3 independent experiments). (**C**) Normalized metabolic activity *evaluated* through the Alamar blue test as a function of the ATP concentration—Linear regression analysis. The ATP contents of HCEC-1CT cells was evaluated at 48 h in a representative subset of 13 wells. Data are shown as individual values with the best-fit curve and its 95% confidence bands (shaded area). (**D**–**H**) Fluorescence images of stained cells (Red: Biotracker 663; Blue: Hoechst) 48 h after treatment with different doses of mitochondria; (**D**) Negative control; (**E**) Mitochondria condition 3.35E10 pt.ml^− 1^; (**F**) Mitochondria condition 7.07E9 pt.ml^− 1^; (**G**) Mitochondria condition 2.83E9 pt.ml^− 1^; (**H**) Mitochondria condition 1.41E9 pt.ml^− 1^. (**I**) Repartition of Biotracker 663’s fluoresce intensity per cell for each condition; p values were calculated using Kruskal–Wallis non-parametric test. To demonstrate experimental consistency across experiments (**A**–**I**), representative data from the same preparation batch (*n* = 13 wells per condition) are shown. Supplementary Fig. 2 provides a comparison between fresh and thawed mitochondrial batches. ****p < 0.0001, ***p < 0.001, **p < 0.01, *p < 0.05; T−: negative control; T+: positive control.

Next, we evaluated the effect of freshly isolated hMSC-derived mitochondria on the metabolic activity of starved HCEC-1CT colon cells assessing their metablic activety via Alamar blue assay, ATP concentration via ATP assay as well as mitochondria content using mitochondria memberane potential indicator, Biotracker red, staining and quantification. Starvation was induced through cultivation in basal medium and the cells were treated with mitochondria suspended in RB. RB alone was used as a negative control. Non-starved cells treated with RB were used as a positive control. The metabolic activity of the cells, assessed through the Alamar blue test was measured at 24 h and 48 h. While no metabolic activity could be detected for the mitochondria alone, Alamar or white DMEM, when normalized to positive control (non-starved cells treated with RB), a remarkable dose-response effect was observed following treatment with freshly-isolated mitochondria of different concentrations of respectively 3.35E10 (p value < 0.01) and 7.07E9 (p value < 0.001) at 24 h (Fig. 2A) or 3.35E10 (p value < 0.001), 7.07E9 (p value < 0.001) and 2.83E9 (p value < 0.01) at 48 h compared to negative control (Fig. 2B). To ensure consistency across experiments, freshly isolated mitochondria from the same preparation batch were used for all in vitro assays (13 wells per condition) in Fig. 2. Interestingly, Alamar results both at 24 h and 48 h significantly correlated with HCEC-1CT ATP contents in a representative subset of 13 wells at 48 h. Metabolic activity as a function of the ATP contents at 24 h and 48 h are shown in Fig. 2C. The Pearson coefficient was of 0.837 with a 95% confidence interval of [0.529; 0.950] at 24 h, and 0.581 with a 95% confidence interval of [0.043; 0.858] at 48 h. Linear regression analysis revealed significant deviation from 0 and non-significant deviation from linearity at both time-points, with a R square of 0.6997 at 24 h and of 0.3370 at 48 h. To assess the mitochondria content of each cell condition after treatment with freshly isolated mitochondria, cells were counterstained with Hoechst and BioTracker 663 red at 48 h. BioTracker 663 red is an indicator of mitochondrial membrane potential commonly utilized in live cell imaging to detect cell viability or metabolic activity (Fig. 2D-H). While the same number of cells were plated to evaluate the metabolic activity of cells exposed to mitochondria with different doses, the cells survival (detected by higher cell density) seemed to be clearly and does-dependently higher for the mitochondria-treated vs. control conditions (Fig. 2D-H). The fluorescence intensity of BioTracker 663 Red, associated to viable mitochondria per cell was measured for wells treated with different mitochondria doses and compared to that of negative control. Results revealed dose-dependent intensities of 4.06 × 10^6^ ± 3.33 × 10^4^ (p value < 0.0001) and 3.21 × 10^6^ ± 2.46 × 10^4^ (p value < 0.0001) at doses of respectively 3.35E10 and 7.07E9 (Fig. 2I). The conspicuous dose-response effect observed with the higher fluorescent intensity for the condition treated with the higher mitochondria dose (and vice versa) is visualized in Fig. 2D-I.

Previous studies have reported that thawed or broken mitochondria are non-viable and ineffective in providing cytoprotection following mitochondrial transplantation^50–52^. To evaluate the viability of transferred mitochondria, we repeated the same in vitro transplantation experiment using frozen–thawed samples (*n* = 2 for thawed mitochondria [MT]) and compared the observed effects with those of freshly isolated mitochondria (*n* = 3 for fresh mitochondria [MF]) across three concentration ranges: [1–8] × 10⁸, [8–70] × 10⁸, and [70–400] × 10⁸ particles/mL. Our results revealed that MT (mean metabolic activity: 0.787 ± 0.052) exhibited a significantly reduced bioenergetic impact compared to MF (mean metabolic activity: 1.024 ± 0.077) in the [8–70] × 10⁸ range (*p* = 0.028), with even more pronounced differences at higher concentrations of [70–400] × 10⁸ particles/mL (MF: 1.5 ± 1 vs. MT: 1.0375 ± 0.05; *p* = 0.001) (Supplementary Fig. 2A). Specifically, a 5.31-fold higher concentration of thawed mitochondria (143.75 × 10⁸ ± 51 × 10⁸ particles/mL) was required to achieve the same metabolic enhancement—defined as a metabolic activity equal to the positive control—as that observed with fresh mitochondria (27 × 10⁸ ± 5 × 10⁸ particles/mL), measured 24 h post-transfer (*p* = 0.028, Supplementary Fig. 2B).

### Post-surgical gastro-cutaneous fistula model in rats

**Fig. 3:**
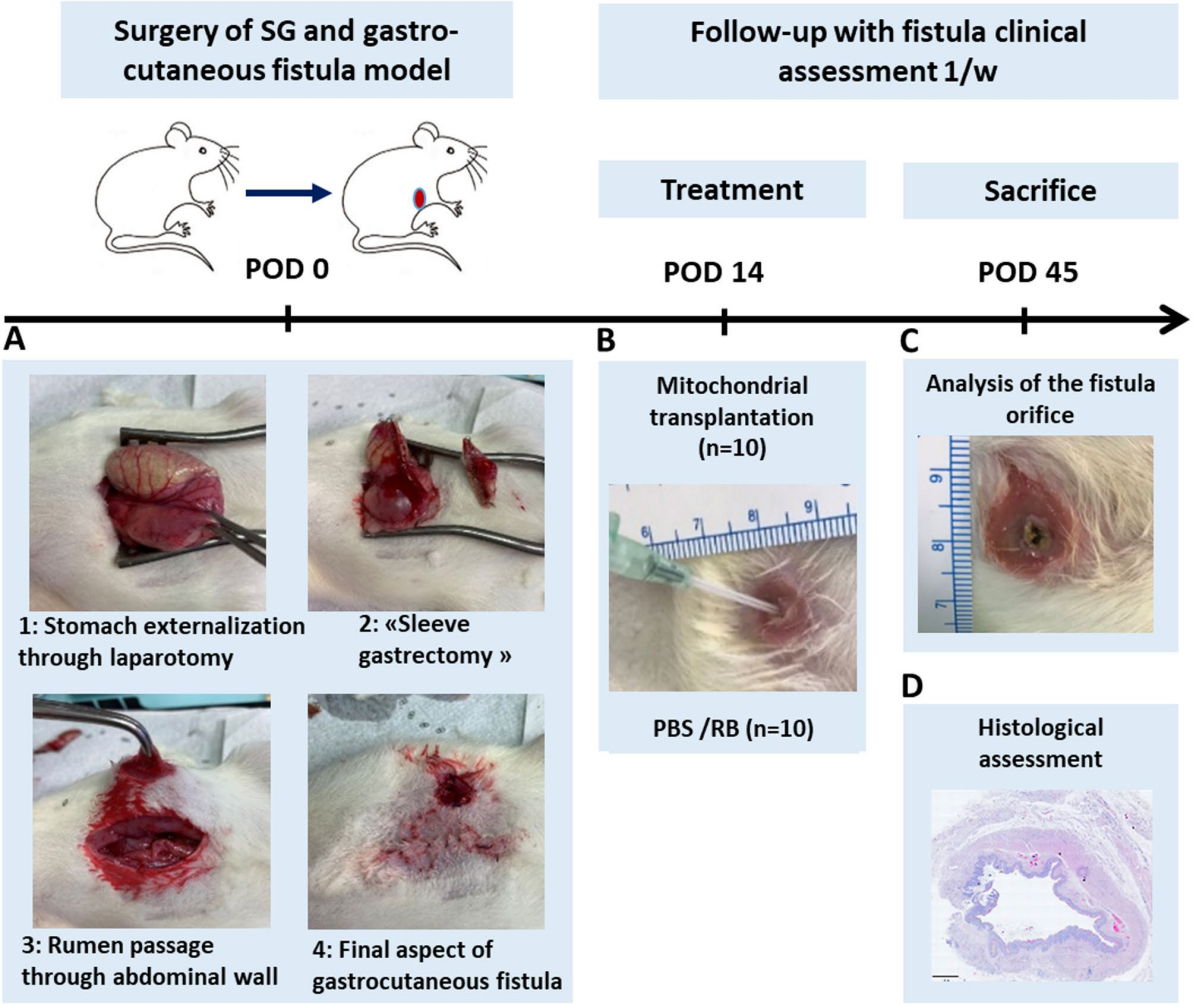
Design of the in vivo experiments. Study timelines and sample size are displayed. (**A**) Surgical procedure for the gastro-cutaneous fistula model induction from laparotomy (step 1), “sleeve gastrectomy” (step 2), rumen passage through abdominal wall (step 3) to final gastrocutaneous fistula formation (step 4) resulting in stomach communication to the skin in rats. (**B**) Percutaneous and intraluminal administration of the mitochondria via the fistula external orifice at days 14 post surgery. (**B**, **C**) Weekly follow-up with clinical assessment of animals and analysis of the fistula orifice diameter over time (at POD 0, POD 14, POD 21, POD 28 and POD 45). (**D**) Histological assessment of the postmortem tissues at POD 45. POD: post-operation day.

A surgical gastro-cutaneous following “sleeve” gastrectomy was performed by gastrostomy at day 0 (experimental design is depicted in Fig. 3). Following the procedure, all fistulas featured an external orifice diameter of larger than 4 mm. There was no complication related to surgery. The mean of weight before surgery was 228.4 +/- 14 g. Evolution of weight during follow-up showed a mean range started from 218.3 +/- 18 g at day 14, right before treatment, to 232.7 +/- 28 g at the end of the follow-up, just before sacrifice. No difference in animal weight was observed between the control and treated groups. All fistulas were permeable (persistent opening) 14 days after the initial procedure with a mean diameter of 4.1 +/- 1.3 mm, indicating the absence of spontaneous closure before treatment.

### Mitochondrial transplantation

**Fig. 4:**
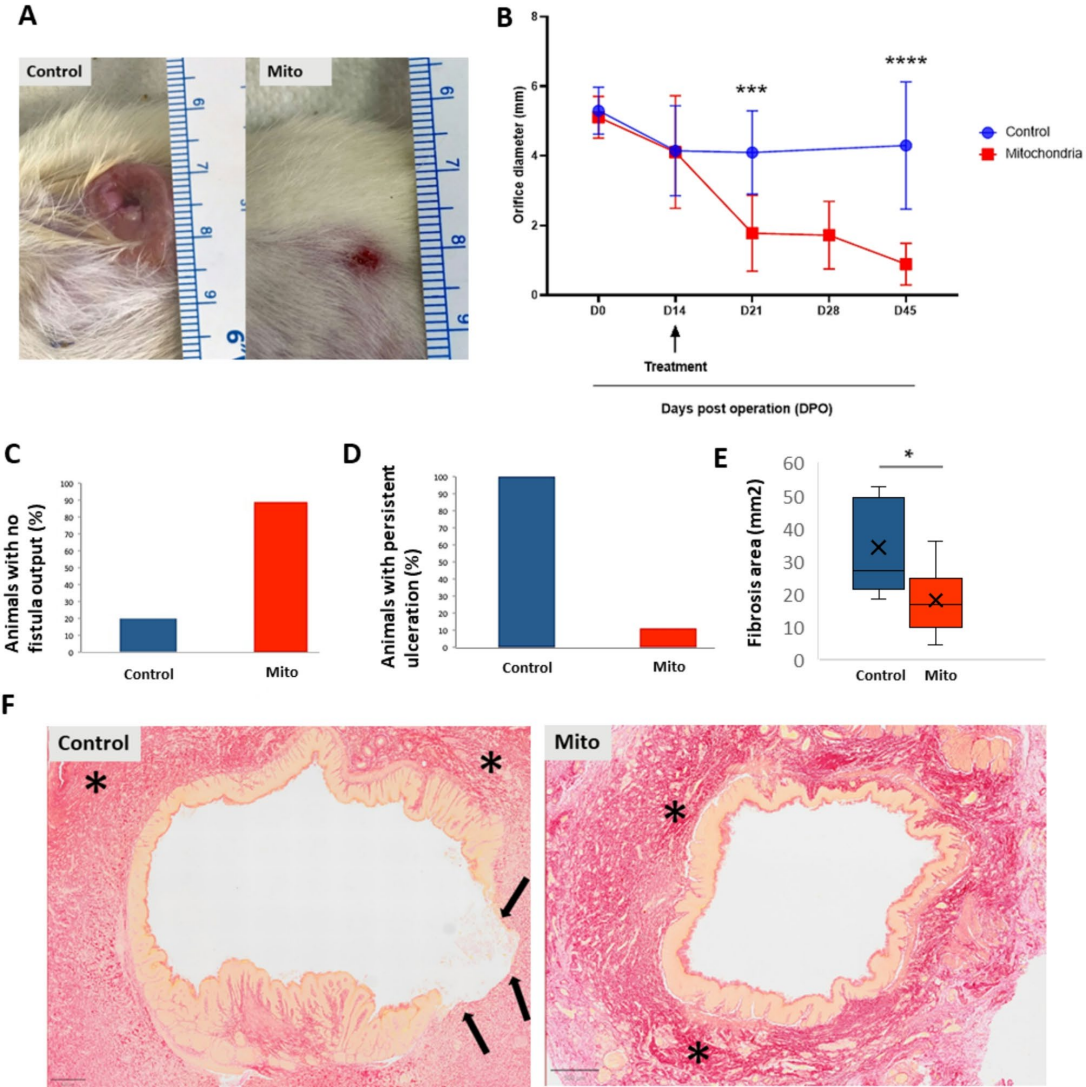
Potency evaluation in vivo following mitochondria transplantation. (**A**) Clinical assessment of the fistula orifice diameter performed on a weekly basis at D14, D21, D28 and morphological correspondence D45 post operation. (**B**) The orifice diameter is significantly reduced over time following local transplantation of hMSC-derived mitochondria (*n* = 9) compared to the control group (*n* = 10, two-way ANOVA, Sidak’s multiple comparisons test). The major reduction in orifice size is observed at D21 corresponding to 7 days after treatment with no significant difference between D21, D28 and D45 (Supplementary Fig. 3). (**C**) Preclinical evaluation at day 45 indicating the percentage of animals per group featuring the absence of feces (no output: 8/9 animals (88.8%) in the mitochondria group vs. 2/10 (20%) in the control group, *p* = 0.0054, Fisher test) at the external fistula orifice as well as percentage of animals per group featuring persistent ulceration (1/9 animals (11%) in the mitochondria group vs. 10/10 (100%) in the control group *p* = 0.0001190, Fisher test) (**D**). Histology analysis (Sirius red staining) showed a decreased fibrosis area in the mitochondrial group (**E**) (34.3 mm^2^ of fibrosis area in the control group vs. 18.3 mm^2^ in the mitochondria group, *p* = 0.03, Mann–Whitney test) with corresponding slices (**F**). The main fibrosis regions in red staining were delimitated by *. The epithelium was identified by an orange staining. Important epithelial damage was observed for the control group and its extent was indicated by black arrows. was used for the statistical analysis of the differences. Error bars represent SEMs. ***p = 0.0003, ****p < 0.0001, *p < 0.05. *DOP* days post operation.

Freshly collected mitochondria suspended in respiration buffer were grafted between 35 and 55 min after sampling in the mitochondrial group at day 14 post operation (14 POD). Control group similarly received PBS (*n* = 5) or respiration buffer (*n* = 5) with no significant difference between them during the entire experiment (at all 5 different clinical evaluation). Animals were clinically evaluated every week following transplantation until the end of study. No evident signs of allergy or toxicity were observed during the entire follow-up. At 21 DPO (7 days post transplantation), although no fistula closure was observed in the animals (Fig. 4A-B), the mean diameter of external orifice was significantly reduced to 1.7 ± 1.09 mm in the mitochondrial group (*n* = 9 animals) versus 4.1 ± 1.19 mm in the control group (*n* = 10 animals, p value < 0.0003). At D45, complete closure of the fistula occurred in 2 cases (22.2% of animals) with the mean orifice diameter of 0.88 ± 0.6 mm (*n* = 9 animals) in the mitochondrial group compared to 4.3 ± 1.82 mm (*n* = 10 animals, *p* < 0.0001) in the control group with 0% complete orifice closure (Fig. 4B). As shown in Fig. 4B, the most significant therapeutic effect (*p* < 0.001) occurred within the first week following mitochondrial transplantation (21 DPO), with no significant differences in orifice diameter between 21, 28, or 45 DPO (Supplementary Fig. 3). The frequency of no fistula output (featuring the absence of feces, Fig. 4C) was significantly (*p* = 0.0054, Fisher test) different when comparing the control group with 20% (2/10 animals) vs. mitochondria group with 88.8% (8/9 animals). Moreover, 8 of 9 cases (88.8%) showed an orifice diameter ≤ 1 mm in the mitochondrial group versus only 1 of 10 (10%) in the control group (*p = 0.001*, Fisher test).

Histological analysis of the post mortem tissues was carried out at 45 POD to assess inflammation and fibrosis (Fig. 4D-F). An extended inflammatory zone surrounding the external orifice was found in the control group in comparison with the mitochondrial group. Qualitative analysis showed the presence of a persistent ulceration (Fig. 4D) in 100% of the control samples (10/10 animals), which was significantly (*p* = 0.0001190, Fisher test) higher than that in the mitochondrial group (observed only in 1/9 animals, 11%). In the fibrosis quantitative analysis, we found that intensity of fibrosis was not different between groups (with 39% (± 7) of fibrosis intensity in the slices of the control group vs. 42% (± 7.5) in the experimental group) but the corresponding area was nearly two times higher in the control group (with 34.3 mm^2^ of fibrosis analysis area in the control group vs. 18.3 mm^2^ in the experimental group, *p* = 0.03, Fig. 4E).

## Discussion

Dysfunctional mitochondria have been implicated in impaired mucosal regeneration and chronic wound formation, underscoring the therapeutic potential of strategies aimed at restoring mitochondrial integrity and bioenergetics, particularly in high-turnover tissues such as the gastrointestinal (GI) tract. Building on this concept, this study explored a novel mitochondria-based cell-free biotherapy derived from human MSCs, demonstrating its efficacy in restoring metabolic function in colonic epithelial cells and promoting fistula closure in vivo. This work contributes to a growing body of evidence supporting mitochondrial transplantation as a next-generation regenerative strategy, and represents the first application of this approach in the context of enterocutaneous wound healing.

As the source of mitochondria, we opted to use human MSCs due to their promising their favorable safety profile, metabolic enrichment, clinical tractability, and regenerative potential in different contexts including gastrointestinal conditions^38,53,54^. Local administration of MSCs combined with PRP has shown promising results in promoting gastric leak closure, both in preclinical studies and a first-in-human clinical series^55,56^. Our results show that structurally intact and viable mitochondria—with conserved membrane potential and high quality—co-expressing 10 structural and functional markers associated with membrane integrity and oxidative phosphorylation complexes, are successfully isolated from hMSCs. MitoTracker Red was used in co-labeling experiments with the mitochondrial outer membrane marker TOM20 to confirm the identity and integrity of freshly isolated mitochondria prior to in vitro and in vivo transplantation. This step validated that the labeled structures were indeed mitochondria. Moreover, the mean diameter of hMSC-derived mitochondria, as measured using ILM, was 366 nm—consistent with previous reports involving muscle cells^57^. In a recent work we demonstrated that ILM, when compared with protein quantification (Bradford assay), TEM (negative staining and cross-section), and TOM20 immunofluorescence, serves as a time-efficient and robust tool for analyzing viable mitochondria for biotherapeutic applications^58^.

Our data show that mitochondria treatment induced a significant dose-dependent effect on the metabolic activity of starved cells. To address if the observed metabolic effect was linked to the energy production, the intracellular ATP content of the HCEC-1CT cells was measured in parallel at similar time points (24 h and 48 h). Interestingly, our data revealed a significant correlation between the dynamic metabolic activity measured by Alamar test (at both time points; 24 h and 48 h) and ATP concentration (at 48 h), that is, the more concentrated mitochondria transplanted the higher is the metabolic activity linked to the energy production revealed by an increase in the levels of cellular ATP. Similar increase in cellular ATP production was reported following exogenous mitochondria transplantation using different recipient cells^59^. Furthermore, to robustly assess mitochondrial uptake, the same treated cells were stained with the mitochondrial membrane marker BioTracker Red—a dye chosen for its high specificity toward functional mitochondria and superior retention during fixed-point imaging. Crucially, staining was performed after evaluating mitochondrial bioactivity through Alamar Blue and ATP assays, ensuring that fluorescence quantification reflected internalized, functionally relevant mitochondria rather than passive dye artifacts. Co-staining with Hoechst enabled precise anatomical localization within recipient cells, and imaging via an automated high-content confocal system (CellInsight CX7 LZR) minimized user bias while providing quantitative consistency across experimental conditions. Notably, the dose-dependent increase observed in metabolic activity (at 24 h and 48 h) and intracellular ATP levels (at 48 h) was paralleled by a corresponding increase in mitochondrial staining intensity, supporting the internalization and integration of exogenous mitochondria by recipient cells. This approach allowed us to correlate the dose-dependent increase in mitochondrial fluorescence intensity with enhanced cellular bioenergetics, reinforcing the functional relevance of mitochondrial uptake. It also addresses recent concerns about dye leakage and misinterpretation in mitochondrial tracking^43^ thereby strengthening the reliability of our uptake findings.

To further investigate the contribution of mitochondrial viability, we compared the bioenergetic effect of freshly isolated versus thawed mitochondria and found a markedly reduced metabolic response in the thawed group, confirming that freeze–thaw cycles compromise viability of transferred mitochondrial. Altogether, these consecutive experimental assays confirm that starved cells dose-dependently enhance their metabolic activity, validated by a marked increase in ATP production via the internalization of transferred mitochondria. Of note, a recent study shows that although freeze–thaw cycles compromise mitochondrial membrane integrity and tricarboxylic acid (TCA) cycle function, electron transport remains functionally intact in frozen mitochondria due to preserved supercomplex activity^60^. This may justify the partial bioenergetic efficacy observed in thawed group. Finally, although the mechanism by which HCEC-1CT cells internalize mitochondria remains to be studied, several evidence show that exogenously delivered mitochondria can be internalized by recipient cells via endocytosis-like mechanisms or by direct interactions with surface receptors such as heparan sulfate proteoglycans (HSPGs)^36,44,61,62^.

Moreover, we demonstrated that direct injection of freshly isolated mitochondria (9.9 × 10¹⁰ particles/mL) in a rat model of post-operative fistula was well tolerated for up to 31 days post-delivery (45 DPO), with no signs of allergic reactions. Notably, this concentration is approximately three times higher than the maximum effective dose used in our in vitro experiments. Our previous studies showed that the therapeutic effects of hMSCs or hMSC-derived EVs in comparable post-surgical fistula models (esophago-cutaneous fistula in pigs or colo-cutaneous fistula in rats) were achieved using local injection of 15–17 million cells (or EV-containing mitochondria) per animal^21,23,63^ —the same number of hMSCs used here for mitochondrial isolation per animal. Our prior quality control analyses confirmed a direct correlation between the number of input cells, mitochondrial particle concentration measured by ILM, and mitochondrial protein content^58^. Moreover, our in vitro data comparing fresh and thawed mitochondrial samples revealed a dose-dependent effect on the metabolic activity of colonic cells, with the best concentration range between 7 and 40 × 10⁹ particles/mL, showing greater benefits at higher concentrations and no significant effect below 8 × 10⁸ particles/mL. McCully et al. (2023) reported that transplanted mitochondria were detectable in myocardial cells, with significant therapeutic effects observed at 2, 4, 8, and 24 h, as well as at 28 days post-delivery, with no adverse effects noted across different doses in a heart ischemia-reperfusion model^61^. Finally, determining the optimal mitochondrial concentration for clinical applications (local or systemic) remains challenging due to the use of varying quantification techniques, which complicates the identification of an effective therapeutic range^42,44,58^.

Our clinical and histological data show a significant decrease in the fistula orifice diameter following hMSCs-derived mitochondria transplantation in 100% of grafted animals with the maximum effect observed at 7 days post transplantation (21 DPO) with no significant difference in orifice diameter between 21, 28 or 45 DPO. Based on this observation and the results of our previous studies using EVs in the same animal model^21^, we have considered 45 days as the final time point for this study. Several in vivo studies have demonstrated rapid and sustained therapeutic effects of locally injected exogenous mitochondria in various animal models. A recent study showed that exogenous mitochondrial transplantation improved survival and neurological outcomes 72 h after resuscitation from cardiac arrest^51^. These findings suggest that the efficacy of mitochondrial grafts can be observed relatively quickly after transplantation. Additionally, a growing body of studies show that mitochondria from human MSCs can enter rodent cells in vitro [reviewed in^64–66^ or in vivo after injection into rodent tissues^65,67–71^.

Applications of isolated mitochondria have shown to be advantageous in the models of spinal cord, lung, kidney and brain injuries^44,72,73^. Moreover, it has been reported that platelet-derived mitochondria transplantation facilitated wound-closure by modulating ROS levels in dermal fibroblasts^74^. Interestingly, Wu et al. (2024) engineered nanomotorized mitochondria with chemotactic targeting capabilities for damaged heart tissue, encapsulated in enteric capsules to protect them from gastric acid erosion prior to oral administration^75^. They discovered that once released in the intestine, the mitochondria were quickly absorbed by intestinal cells and entered the bloodstream, indicating their rapid uptake by the intestinal cells and distribution in vivo. The results of this study demonstrate, for the first time, that local injection of mitochondria can remarkably promote the closure of post-operative fistulas in 100% of transplanted animals in 7 days.

While the underlying mechanisms behind this rapid and efficient therapeutic effect are still under investigation, our in vitro data—using the same batches of freshly isolated mitochondria as those used for in vivo transplantation—demonstrate a clear dose-dependent increase in BioTracker Red⁺ mitochondria, correlated with enhanced metabolic activity and elevated ATP content in colonic recipient cells, indicating mitochondrial internalization as a likely driver of functional recovery in postoperative fistula model. Masuzawa et al. observed that mitochondrial transplantation led to the internalization of mitochondria by cardiomyocytes, resulting in enhanced ATP production and an increase in differentially expressed proteins related to mitochondrial pathways and cellular respiration^76^. The molecular mechanisms underlying hMSC-derived mitochondrial delivery across various diseases have been comprehensively reviewed in recent literature^34,77^. While the results of this study are encouraging, future investigations should aim to elucidate the delivery mechanisms and post-transplantation fate or biogenesis of exogenous mitochondria in digestive fistula.

## Conclusions

This study establishes, for the first time, that local mitochondrial transplantation can rapidly and effectively close post-operative digestive fistulas, achieving complete closure in all treated animals within just seven days. By harnessing structurally intact, functionally potent mitochondria isolated from human MSCs, we demonstrate a powerful cell-free regenerative strategy that restores epithelial bioenergetics and promotes tissue repair. Our findings not only validate the feasibility, safety, and efficacy of mitochondrial therapy in gastrointestinal wound healing, an essential step for clinical translation. These results position mitochondrial transplantation as a next-generation biotherapy for high-energy demanding tissue injuries—transforming our approach to regenerative medicine in the gastrointestinal tract and beyond. Future studies deciphering the fate, trafficking, and long-term integration of grafted mitochondria will be critical to fully unlock their therapeutic potential.

## Methods

### Human adipose tissue-derived mesenchymal stem cells (hMSCs) culture

Human adipose tissue-derived MSC (CellEasy batch number N°9297) were grown in T175 culture flasks using α-Minimal Essential Medium (α-MEM) supplemented with 10% fetal bovine serum (FBS) at 37 °C, 5% CO_2_ with 20 mL of media utilized to ensure complete flask coverage. The medium was refreshed every 3 days, and cells were grown until reaching 90% confluence before passaging.

To detach the cells, the culture medium was aspirated, and the flasks were washed with phosphate-buffered saline (PBS, Gibco, Thermofisher). The washed cells were then incubated with 0.05% trypsin ethylenediaminetetraacetic acid (EDTA, Gibco, Thermofisher) for 2 to 3 min at 37 °C with 5% CO_2_. Mechanical detachment was facilitated by tapping the flasks. Following trypsinization, an equal volume of α-MEM with 10% FBS was added to neutralize the trypsin. The cell suspension was centrifuged at 300 g for 10 min, and the supernatant was discarded. Cell quantification and viability assessment were performed using the NucleoCounter^®^ NC-200™ automated cell analyzer (NC-200, Chemometec). Only cells from passages 4 to 8 were utilized for mitochondria isolations.

### Isolation of mitochondria from hMSCs

A commercial mitochondrial isolation kit for cultured cells (ab110170, Abcam)^78–81^ was used to isolate mitochondria from hMSCs. Initially, cells were pelleted via centrifugation at 300 g, followed by rapid freezing and thawing to weaken cell membranes. Subsequently, the cells were suspended in Reagent A at a concentration of 5.0 mg protein/ml (approximately 1 ml per 25 million cells) and incubated on ice for 10 min. Cell disruption was achieved using a sonication probe (Sonicator FB50, Fisher Scientific), with ultrasound applied in 3 cycles of 10-second bursts at 20% power, separated by 10-second intervals (total active phase of 30 s), while keeping the cell suspensions cooled on ice.

Next, the homogenate underwent centrifugation at 1,000 g for 10 min at 4 °C to remove remaining cells, cell debris, and nuclei. The resulting supernatant (SN) #1, containing mitochondria, was collected, while the pellet was resuspended in Reagent B to the same volume as Reagent A. The cell rupturing step was repeated to release mitochondria from the remaining intact cells and the resulting supernatant SN #2 was collected, while the pellet was discarded. SNs #1 & #2 were combined, thoroughly mixed, and centrifuged at 12,000 g for 15 min at 4 °C. The resulting pellet constituted the isolated mitochondrial mass was resuspended in ice-cold respiration buffer (RB): 250 mM sucrose, 5mM KH_2_PO_4_, 10 mM MgCl_2_, 20 mM HEPES (pH 7.2) and 1 mM EGTA (pH 8); quantified, dose adjusted, aliquoted and kept on ice until transplantation. For mitochondria characterization, the pellet was washed with Reagent C supplemented with Protease Inhibitors (11697498001, Roche), and aliquoted for the subsequent characterization, in vitro or in vivo experiments or stored at -80 °C until the mitochondrial quality assays were conducted.

### Visualization of isolated mitochondria

MitoTracker Red CMXROS is a red-fluorescent dye that selectively stains intact mitochondria with its accumulation contingent upon mitochondrial membrane potential. Viability of freshly isolated mitochondria was assessed using MitoTracker Red CMXROS (M7512, Invitrogen) staining at 200 nM for 40 min followed by two washes with PBS 1X and immediate observation of mitochondria (resuspended in 10 µL in PBS, mounted onto slides) using fluorescence microscopy (EVOS M5000, ThermoFisher).

### Immunofluorescence imaging of isolated mitochondria

Immunofluorescence imaging of isolated mitochondria involved characterizing freshly isolated samples through mitochondria-specific immunostaining (following or not MitoTracker Red CMXROS staining). Initially, isolated mitochondria were incubated either with an Alexa Fluor^®^ 488 Anti-TOMM20 (translocase of outer mitochondrial membrane 20 homolog) antibody [EPR15581-39] - Mitochondrial Marker (ab205486, abcam) or with a primary rabbit anti-mouse anti-TOM20 antibody (11802-1-AP, Proteintech) for 30 min followed by 30 min incubation in the dark with a secondary antibody (Goat anti-Rabbit IgG) conjugated with green fluorescent AlexaFluor 488 (A-11008, ThermoFisher). TOM20 antibody specifically binds to the TOM20 complex located in the mitochondrial outer membrane. Samples were washed twice with PBS 1X and underwent centrifugation (12000 g, 15 min, 4 °C) at each washing step. Visualization and imaging were achieved using a Cell Insight CX7 LZR automated confocal spinning-disk fluorescence microscope (ThermoFisher Scientific) or EVOS M5000 microscope (ThermoFisher).

### Western blot analysis of mitochondria protein markers

Mitochondria obtained from hMSCs underwent homogenization and lysis in ice-cold 30 mM Tris-EDTA buffer, pH 7.2, supplemented with 1 mM DTT, 1% (v/v) Triton X-100, 10% (w/v) anti-phosphatase cocktail, and 14% (w/v) anti-protease cocktail (11697498001, Roche), for 30 min on ice. Homogenate was gently mixed on an agitating plate for 2 min at ice-cold temperatures. The proteins were quantified using a Bradford assay. From each sample 20 µg of total protein was loaded and separated on 4–20% Mini-PROTEAN^®^ TGX™ Precast Protein Gels (Bio-Rad), followed by electroblotting onto membranes of Trans-Blot Turbo Midi 0.2 μm PVDF Transfer Packs (Bio-Rad). Subsequently, the membranes were rinsed in TBS-Tween 20 buffer (TBST) and blocked for 1 h in Every-Blot Blocking Buffer (BioRad) before being washed with TBST.

For antibody incubation, the membranes were then incubated overnight at 4 °C with either: (i) rabbit anti-TOM20 (1:5000, 11802-1-AP, ThermoFisher); (ii) total oxidative phosphorylation (OXPHOS) Human WB Antibody Cocktail (MS601) (1:700, ab110411, Abcam); (iii) Membrane Integrity WB Antibody Cocktail (MS620) (1:250, ab110414, Abcam); or (iv) rabbit anti-GAPDH antibody (1:2500, ab9485, Abcam) as a loading control. Following washing, the blots were incubated with the corresponding goat anti-rabbit horse-radish peroxidase (HRP)-linked Antibody (1:2000, 7074, Cell Signaling) or goat anti-mouse HRP-conjugated secondary antibody (1:2000, 7076, Cell Signaling) for 2 h at room temperature.

Peroxidase activity was revealed using a chemiluminescent detection kit (ECL Plus substrate, GE Healthcare). Protein expression visualization and acquisitions were performed using the Syngene PXi (UK), with documentation accomplished using GeneSys image capture software (UK). Post-spin supernatant was loaded as a control at 20 µg for all the experiments.

### Interferometric light microscopy (ILM) measurements of isolated mitochondria using videodrop

Measurements of isolated mitochondria using ILM with Video-Drop technology were conducted to determine their concentration and average hydrodynamic radius. Drops of 7 µL each were dispensed onto a round cover slip using a micropipette, followed by positioning the stage towards the objective. The QVIR 2.6.0 software (Myriade, Paris, France) was employed to illuminate the sample using a 2 W blue LED light.

The concentration of particles was determined by the number of detected particles within the measured volume, which is influenced by both microscope parameters and particle size. The hydrodynamic radius (size) was estimated by tracking the motion of imaged particles in recorded movies, as they undergo thermal agitation. All detected particles contribute to the number density of particles per milliliter (concentration).

Tracking was initiated with a minimum of 300 particles, with up to 50 videos analyzed, each containing 100 frames. A minimum saturation of 92% was considered, and a relative threshold of 4.2, as specified by the manufacturer, was applied for detection. Macroparticle detection was enabled by setting a minimum radius of 10 and a minimum number of hot pixels of 150, along with drift compensation. To ensure accuracy, signals from PBS 1X were monitored and consistently found to be below the detection limit.

### Electron microscopy

#### Scanning electron microscopy (SEM)

Samples were mounted on aluminum stubs (32 mm diameter) with carbon adhesive discs (Agar Scientific, Oxford Instruments SAS, Gomez-la-Ville, France) and visualized by field emission gun scanning electron microscopy (SEM FEG) as secondary-electron images (2 keV, spot size 30) under high-vacuum conditions with a Hitachi SU5000 instrument (Milexia, Saint-Aubin, France). SEM analyses were performed at the Microscopy and Imaging Platform MIMA2 (INRAE, Jouy-en-Josas, France) DOI: MIMA2, INRAE, 2018. Microscopy and Imaging Facility for Microbes, Animals and Foods.

#### Transmission electron microscopy (TEM)—negative-staining

Isolated mitochondria (5 µL) were applied directly onto a carbon film membrane on a 300-mesh copper grid, followed by staining with 1% uranyl acetate solution dissolved in distilled water. The grids were subsequently air-dried at room temperature. Examination of the grids was performed using a Hitachi HT7700 electron microscope operating at 80 kV (Elex-ience), and images were captured with a charge-coupled device camera (AMT).

#### Transmission electron microscopy (TEM)—cross-sectioned method

Mitochondrial suspensions (45 µL) were subjected to centrifugation at 12,000 ×g for 15 min, after which the supernatant was carefully removed, and the resulting pellets were fixed with 2% glutaraldehyde in 0.1 M sodium cacodylate buffer at pH 7.2 for 1 h at room temperature. Following removal of the supernatant, the pellets underwent three washes in PBS 1X. Subsequently, samples were contrasted with 0.2% Oolong Tea Extract (OTE) in cacodylate buffer, postfixed with 1% osmium tetroxide containing 1.5% potassium cyanofer-rate for 1 h. After an additional three washes in PBS 1X, the pellet was dehydrated through a graded series (30–100%) and gradually substituted in a mixture of ethanol-epon, finally being embedded in 100% Epon (Delta microscopie, France) over a 24-hour period. The pellet underwent a final embedding in Epon, cured at 37 °C overnight, and further hardened at 65 °C for two additional days.

Ultrathin Sections of 80 nm each were then collected onto 200-mesh copper grids, stained with 2% uranyl acetate in 50% methanol for 10 min, and counterstained with 1% lead citrate for 7 min. The grids were examined using a Hitachi HT7700 electron microscope operated at 80 kV (Milexia – France), and images were acquired with a charge-coupled device camera (AMT). This analysis was conducted at MIMA2 MET – GABI, INRA, Agroparistech, 78352 Jouy-en-Josas, France.

### In vitro evaluation of the effects of mitochondria on the metabolic activity of starved HCEC-1CT cells

Cells of the HCEC-1CT colon epithelial progenitor cell line were obtained from Evercyte. This cell line was immortalized from non-tumoral adult human colon biopsies. HCEC-1CT were routinely cultivated at 37 °C with 5% CO_2_ in ColoUP medium, composed of four parts DMEM (Gibco, 31966-021) and one part M199 (Gibco, 31150-022), 2% Cosmic Calf Serum (Hyclone, SH30087), 20 ng/ml hEGF (Sigma, E9644), 10 µg/ml Insulin (Sigma, I9278), 2 µg/ml Apotransferrin (Sigma, T2036), 5 nM Sodium Selenite (Sigma, 214485), 1 µg/ml Hydrocortisone (Sigma, H0396). Cells from passages 10 to 58 were utilized for the subsequent experiments.

HCEC-1CT cells were seeded in 96 well plates (VWR 734–2327) at 5000 cells per well, then amplified for 48 h in ColoUp medium. Differentiation was induced by 120 h culture in a differentiation medium composed of four parts DMEM and one part M199, 0.1% Cosmic Calf Serum, 1.25ng/ml hEGF, 10 µg/ml Insulin, 2 µg/ml Apotransferrin, 5nM Sodium Selenite, 1 µg/ml Hydrocortisone, 5µM 6-bromoindirubicin-3-oxime / GSK-3 inhibitor (Sigma, 361550-1MG) and 100 µg/ml Primocin (InVivogen, ant-pm-05).

After differentiation, the cells were washed and starvation was induced by shifting to basal medium composed of four parts DMEM and one part M199 with no further supplementation. Starvation is known to modify the cellular metabolic activity in vitro^55^. Three independent experiments were performed to isolate fresh-mitochondria from hMSCs. HCEC-1CT cells were then treated for 48 h with either freshly-isolated hMSC mitochondria suspended in RB (at different doses of 10^8^-3.33 × 10^10^ particles/mL in the well, 13 wells per condition) or an equivalent volume of RB (negative control, 11 wells/experiment). The positive control group was cultivated in fresh differentiation medium instead of basal medium, and treated with RB (11 wells/experiment). Metabolic activity of the starved HCEC-1CT cells treated or not with freshly-isolated mitochondria (three independent batches) was evaluated at 24 h and 48 h through the Alamar Blue Assay (Invitrogen, DAL1100). To analyze the metabolic activity, in brief, cells were first washed in PBS, then incubated for 3 h in Alamar blue reagent diluted 10 folds in white DMEM (Gibco, 31053-028). Mitochondria alone in well without cells (with highest concentration in each series of experiment), Alamar (white DMEM + 10% Alamar in well without cells (served as blank for analysis)) or white DMEM (in wells without cells) were used as extra controls (3 wells each/experiment). Metabolic activity was then estimated by the rate of reduction of resazurin into resorufin in viable cells as evaluated through fluorescence measurements (540/590nm, integration time 400ms, SpectraMax^®^ iD3 Microplate Reader, Molecular Devices). Similar experiments (*n* = 2 preparations) were performed using mitochondria that had been stored at − 80 °C for several weeks and then thawed.

To further verify the cell metabolic activity following exposure to isolated mitochondria, the ATP content of a subset of wells (13 wells per condition) was measured using the ATP Bioluminescence Assay Kit (Abcam ab113849) according to the supplier’s instructions. We then evaluated whether the cells’ ATP contents at 48 h correlated with their metabolic activity measured by the Alamar blue assay through computation of Pearson’s correlation coefficient after checking whether the assumption of normality could reasonably be made then linear regression analysis, using the GraphPad Prism software (version 8.0.2 and 10.0.2).

Mitochondria staining was performed to visualize mitochondria content of the HCEC-1CT cells 48 h after in vitro treatment. Briefly, the cells were washed and stained with Hoechst at 200nM and Bio Tracker™ 633 Red Mitochondria Dye at 100nM (Sigma-Aldrich SCT137) for 30 min at 37 °C. The cells were then washed and kept in PBS. Sixteen fields per well and one well per condition was imaged with a Cell Insight High-Content Screening CX7 LZR automated confocal spinning-disk fluorescence microscope (Thermo-Fisher Scientific) at 20X magnification to minimize quantification bias.

### Rat model of gastro-cutaneous fistula

All experiments were approved by the Animal Care and Use Committee in France and the Ministry of Higher Education and Research (APAFIS #35921-2022010121242601). This study is reported in accordance with the ARRIVE guidelines (https://arriveguidelines.org) for the reporting of animal research. A total of 20 female 11-week-old Wistar rats (purchased from JANVIER LABS, Le Genest-Saint-Isle, France) were divided into 2 groups: a control group (*n* = 10), an experimental group (*n* = 10) with a mean weight of 221 g (range 212–235 g). Minimization method was used to ensure groups were comparable in terms of gender, weight and age. The sample size was determined based on our previous studies evaluating the therapeutic potential of hMSCs-derived EVs or hMSCs in post operative fistula healing model^21^.

The animals went through a 7-day acclimatization period with water and food ad libitum. They were housed in the laboratory animal room, in cages, with regulated temperature, ventilation, and respecting light–dark cycles. Anesthesia was performed under 2% of isoflurane and analgesia was induced with 0.05 mg/kg of buprenorphine. After a midline laparotomy of 2–3 cm, the entire greater curve of the stomach was identified. Pylorus and esogastric junction were identified and gastrosplenic ligament sectioned. The “sleeve” gastrectomy was performed using a linear stapler, from 5 mm of the pylorus to the distal part of the rumen, carefully avoiding stenosis of the remnant gastric tube. The upper part of the rumen was passed with a staggered opening through the abdominal muscle and the subcutaneous space, forming a 5 mm-long tract. Four stitches (PDS 4/0) were used to attach the stomach to the skin at the site of the incision on the left flank of the rat, creating a 5 mm large gastro-cutaneous fistula model. Postoperative analgesia was performed.

### Mitochondria transplantation

Fourteen days after surgery, animals (*n* = 10) were anesthetized using 2% isoflurane, and were locally injected at the lesion site with 0.6 mL of freshly isolated mitochondria (9.9 × 10^10^ particle/mL per rat, equal to 800 µg mitochondria protein, generated by 17 × 10^6^ hMSCs) resuspended in mitochondria respiration buffer. Each animal received 4 local injections of 100 µL each of fresh mitochondria (around the external orifice of fistula plus 200 µL injected directly inside the fistula tract wall). Mitochondria from three independent isolation were used. Control group similarly received 0.6 mL of PBS or respiration buffer (*n* = 10). With one animal in the control group died following the surgery, 19 animals (in total) were finally followed-up until the end of the experiments.

### Preclinical follow-up

Rats (*n* = 10 for Mito vs. *n* = 9 for control groups) were daily followed up to 45 days post operation (DPO) and clinically assessed at DPO 0, DPO 14, DPO 21, DPO 28 and DPO 45 corresponding to 30 days after treatment). Animals were anesthetized every week, using 2% isoflurane, for fistula measures using a graduated ruler, and pictures were taken for external control. The presence of feces at the fistula orifice, indicative of positive fistula output, was evaluated macroscopically. The absence of feces indicated negative fistula output.

### Histological analyses

At D60, rats were sacrificed, under 2% isoflurane anesthesia, using intracardial injection of thiopental. The fistula site as well as its periphery (1 cm length, 2 specimens each per rat) were collected and transferred to a formalin solution. Specimens were embedded in paraffin and sectioned perpendicular to the center of the fistula to obtain thin tissue sections of 7 μm, which were stained with hematoxylin and eosin (HE) and Sirius Red (fibrosis assessment). Slices were analyzed with an optical microscope (Leica DMIL). Histological analysis was performed by an external pathologist (CB), blinded to treatment allocation. All slices were also digitally scanned (Digitiser Hamamatsu Photonics^®^, Massy, France) and analyzed with dedicated software (Halo) for fibrosis quantitative analysis.

### Statistics

The results were presented as means ± standard deviation for continuous variables, and as percentages for categorical variables. Fischer’s exact test was carried out for comparisons between categorical variables and the nonparametric Mann–Whitney test was used for non-paired continuous variables. An estimation of the p value by the Chi-square test was carried out for the comparison concerning the number of cases per group. Pearson’s correlation coefficient was calculated, and linear regression analysis was conducted for the in vitro assessment of ATP content and Alamar Blue metabolic activity. A two-way ANOVA, followed by Sidak’s multiple comparisons test, was employed to evaluate differences in the fistula orifice diameter over time between the treated and control groups. Statistical analysis was conducted using SAS version 9.1 (SAS Institute, Cary, NC, USA) or GraphPad Prism v.8.0.2 software (Graphpad Software, La Jolla, CA, USA). The notations for the different levels of significance are indicated as follows: *p < 0.05, **p < 0.01, ***p < 0.001, ****p < 0.0001. For all the experiments, a p-value inferior to 0.05 is considered significant.

## Supplementary Information

Below is the link to the electronic supplementary material.

Supplementary Material 1

## Data Availability

The datasets used and/or analyzed during the current study are available from the corresponding authors on reasonable request.

## Abbreviations

hMSCs: Human mesenchymal stem/stromal cells
HCEC-1CT: Human colonic epithelial cells
EV: Extracellular vesicles
ECMO: Extracorporeal membrane oxygenation
MHC: Major histocompatibility complex
α-MEM: α-Minimal Essential Medium
FBS: Fetal bovine serum
PBS: Phosphate-buffered saline
EDTA: Ethylenediaminetetraacetic acid
SN: Supernatant
Mito: Isolated mitochondria
ILM: Interferometric light microscopy
Part/mL: Particles per milliliter
SEM: Scanning electron microscopy
TEM: Transmission electron microscopy
OXPHOS: Oxidative phosphorylation
POD: Post-operative day(s)
RB: Respiration buffer
LSG: Laparoscopic sleeve gastrectomy
PRP: Platelet-rich plasma
