# Supplementary material for: Therapeutic potential of human mesenchymal stromal cell-derived mitochondria in a rat model of surgical digestive fistula
Source: Sci Rep. 2025 Aug 9;15:29167. doi: 10.1038/s41598-025-13887-3 (PMC12335506; doi:10.1038/s41598-025-13887-3)
Supplement: Supplementary file 1 — Supplementary Material 1 [file 41598_2025_13887_MOESM1_ESM.docx]

**Therapeutic potential of human mesenchymal stromal cell-derived mitochondria in a rat model of surgical digestive fistula**

Antoine Mariani^1^, Augustin Guichard^2^, Anna C. Sebbagh^2^, André Cronemberger Andrade^2^, Zahra Al Amir Dache^2^, Christopher Ribes^2^, Dmitry Ayollo^2^, Mehdi Karoui^1^, Gregory Lavieu^2^, Florence Gazeau^2^, Amanda K. A. Silva^2,^ *, Gabriel Rahmi^3,^ * and Sabah Mozafari^2, 4,^ *

1. Assistance Publique-Hôpitaux de Paris, Service de chirurgie digestive et oncologique, Hôpital Européen Georges Pompidou, Paris 75015, France.
2. Université Paris Cité, CNRS UMR8175, INSERM U1334, Laboratory NABI (Nanomédecine, Biologie Extracellulaire, Intégratome et Innovations en santé), Paris 75006, France.
3. Assistance Publique-Hôpitaux de Paris, Service d'hépato-gastro-entérologie et endoscopies digestives, Hôpital Européen Georges Pompidou, Paris 75015, France.
4. Department of Clinical Neurosciences and National Institute for Health Research (NIHR) Biomedical Research Centre, University of Cambridge, Cambridge CB2 0AH, UK.

* These authors jointly supervised this work:

[amanda.brun@u-paris.fr](mailto:amanda.brun@u-paris.fr)

[gabriel.rahmi@aphp.fr](mailto:gabriel.rahmi@aphp.fr)

[sm3010@cam.ac.air](mailto:sm3010@cam.ac.air)

**
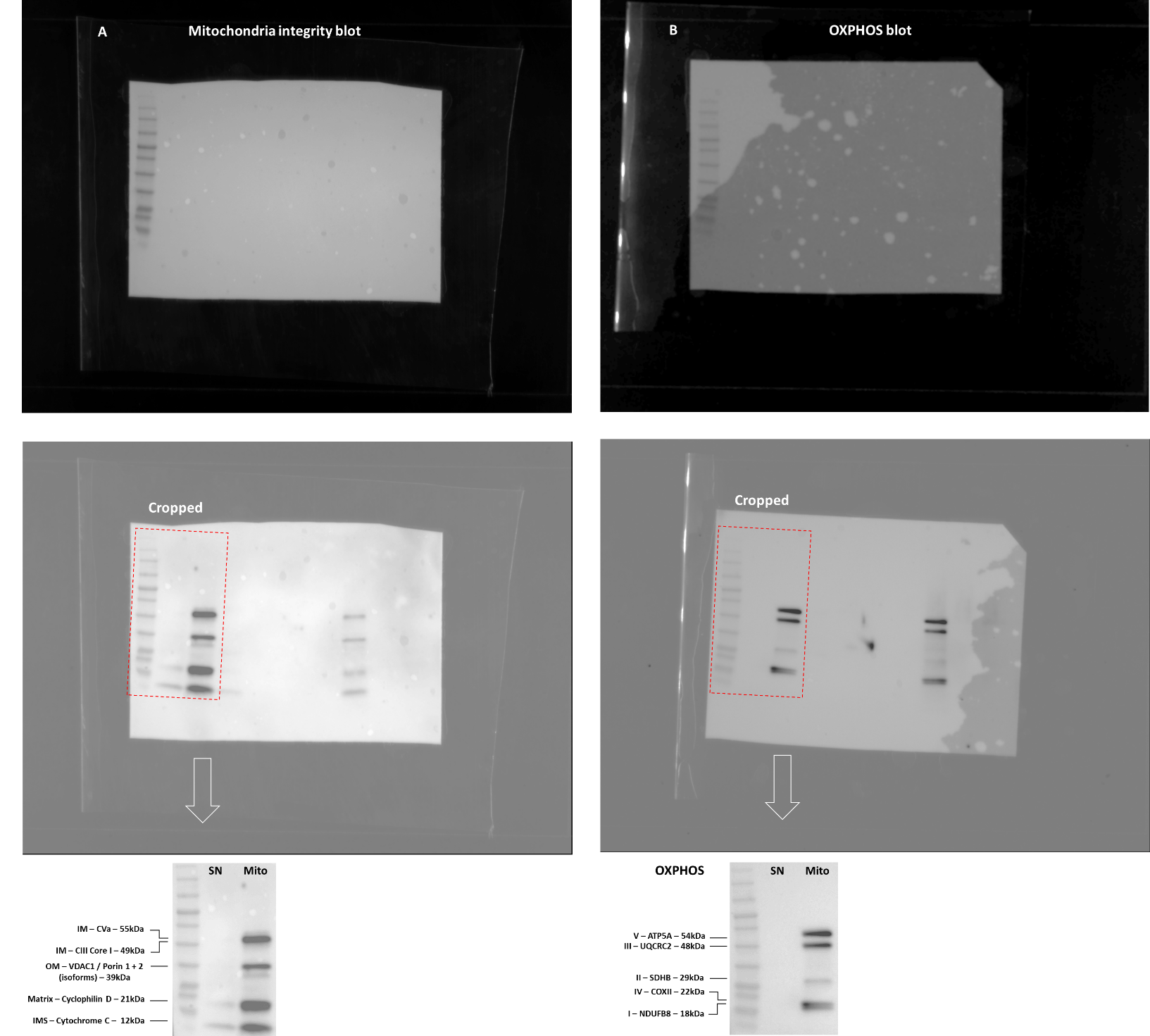
Supplementary Figure 1**

***Supplementary Figure 1.*** *The uncropped full-length blots. To improve the clarity and conciseness of the presentation the blots of the mitochondria membrane integrity as well as OXPHOS western blotting were cropped. (A and B) shows these uncropped images before and after immunodetection. The cropped blots containing the band sizes and labels are found in the lower panels as well as in the Figure 1F-G.*

*
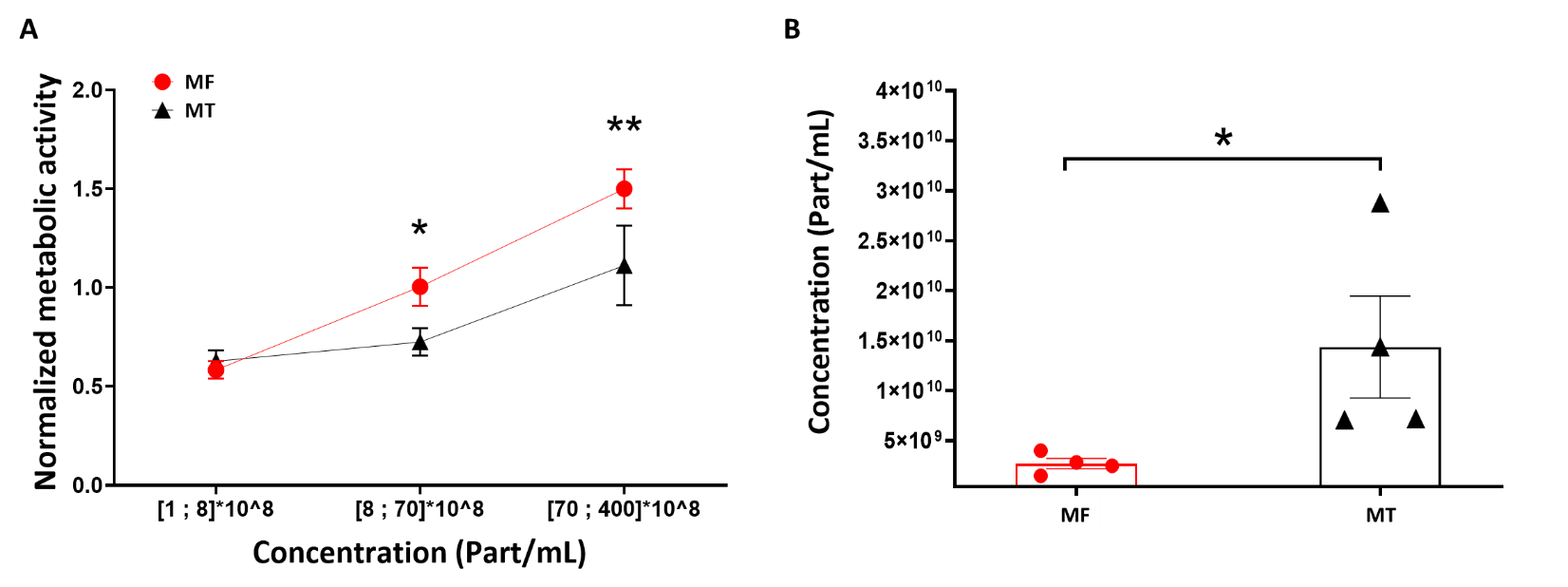
*

***Supplementary Figure 2. Freshly isolated mitochondria induce greater metabolic activity in recipient cells.*** *To assess the viability of transferred mitochondria, in vitro transplantation assays were repeated using frozen–thawed mitochondria (MT) and compared to freshly isolated mitochondria (MF). (A) Thawed mitochondria exhibited significantly reduced metabolic activity at concentrations above 8 × 10⁸ particles/mL (n = 2 for MT vs. n = 3 for MF; p = 0.028), with a more pronounced reduction observed at concentrations exceeding 70 × 10⁸ particles/mL (p = 0.001). (B) A 5.31-fold higher concentration of thawed mitochondria was required to achieve the same metabolic enhancement—defined as normalized metabolic activity equivalent to the positive control—as fresh mitochondria, measured 24 hours post-transfer (p = 0.028). Two-way ANOVA, Sidak's multiple comparisons test in A. Two-tailed Mann–Whitney test in B. Error bars represent SEMs. * p < 0.05, ** p < 0.01.*

***Supplementary Figure 3. Therapeutic effect of mitochondrial transplantation plateaus after the first week.*** *The most significant reduction in fistula orifice diameter was observed within the first week following mitochondrial transplantation (measured at 21 days post-operation, DPO). No significant differences in orifice diameter were detected between 21, 28, or 45 DPO, indicating a plateau in therapeutic efficacy after the initial week. Two-way ANOVA, Sidak's multiple comparisons test (n=9). Error bars represent SEMs. *** p < 0.001.*
